# Determination of nitrogen balance in agroecosystems

**DOI:** 10.1016/j.mex.2017.06.001

**Published:** 2017-07-03

**Authors:** Upendra M. Sainju

**Affiliations:** USDA, Agricultural Research Service, Northern Plains Agricultural Research Laboratory, Sidney, MT 59270, USA

**Keywords:** Determination of nitrogen balance in agroecosystems, Nitrogen cycling, Nitrogen management, Environmental sustainability, Crop productivity, Agricultural practices

## Abstract

Nitrogen balance in agroecosystems provides a quantitative framework of N inputs and outputs and retention in the soil that examines the sustainability of agricultural productivity and soil and environmental quality. Nitrogen inputs include N additions from manures and fertilizers, atmospheric depositions including wet and dry depositions, irrigation water, and biological N fixation. Nitrogen outputs include N removal in crop grain and biomass and N losses through leaching, denitrification, volatilization, surface runoff, erosion, gas emissions, and plant senescence. Nitrogen balance, which is the difference between N inputs and outputs, can be reflected in changes in soil total (organic + inorganic) N during the course of the experiment duration due to N immobilization and mineralization. While increased soil N retention and mineralization can enhance crop yields and decrease N fertilization rate, reduced N losses through N leaching and gas emissions (primarily NH_4_ and NO_x_ emissions, out of which N_2_O is a potent greenhouse gas) can improve water and air quality.

• This paper discusses measurements and estimations (for non-measurable parameters due to complexity) of all inputs and outputs of N as well as changes in soil N storage during the course of the experiment to calculate N balance.
• The method shows N flows, retention in the soil, and losses to the environment from agroecosystems.
• The method can be used to measure agroecosystem performance and soil and environmental quality from agricultural practices.

## Method details

### Nitrogen balance

Nitrogen balance is measured by deducting N outputs and changes in soil total N storage from N inputs [1], [2] as follows:

(1) Nitrogen balance = N inputs − N outputs − changes in soil total N

(2) Where N inputs = N fertilization from inorganic N fertilizers + N fertilization from manures and amendments + atmospheric N depositions (rain, snow, and dry deposition) + biological N fixation (symbiotic + non-symbiotic N fixation) + irrigation water + crop seed.

(3) N outputs = crop N removal (grain and biomass) + N losses (through N leaching, NH_4_ volatilization, denitrification, gas emissions [NO_x_], surface runoff, soil erosion, and plant senescence)

(4) Changes in soil total N = Soil total N at the end of the experiment − soil total N at the beginning of the experiment.

A positive value of N balance indicates that N is gained in the system and negative value indicates loss. When all sources, sinks, and losses of N are accounted, there should be no net gain or loss of N if N is recycled efficiently. This, however, occurs rarely due to various factors, such as variations in soil and climatic conditions, N management, soil and crop management practices, and difficulty in measurement of some parameters, such as atmospheric N depositions, biological N fixation, and N losses through various processes. Step-by-step measurement or estimation of each parameter for N balance is described below and calculation is shown in Table 1.

**Table 1:** Nitrogen balance due to difference between total N inputs and outputs and soil total N at the beginning and end of the experiment.

Source	Value
	kg N ha^−1^
N inputs	
Total N fertilization rate	A
Total manure application	B
Symbiotic N fixation	C
Atmospheric N deposition	D
Irrigation water	E
N added by crop seed	F
Non-symbiotic N fixation	G
Total N input	X = A + B + C + D + E + F + G

N outputs	
Grain and/or biomass N removal	I
Denitrification N loss	J
Ammonia volatilization N loss	K
N loss at plant senescence	L
Gaseous N loss (other than NH_3_ volatilization)	M
N loss due to surface runoff	N
N leaching	O
N loss due to soil erosion	P
Total N output	Y = I + J + K + L + M + N + O + P

Soil total N	
Soil total N at the beginning of the experiment	Q
Soil total N at the end of the experiment	R
Change in soil total N	Z = R − Q

N balance	
N balance during the experiment duration	N_b_ = X − Y − Z
N balance per year (kg N ha^−1^ yr^−1^)	N_b_/No. of years of the experiment duration

### Soil total nitrogen

Changes in soil total N at the beginning and the end of the experiment after accounting for all N inputs and outputs from agroecosystems indicate if soil is a sink or source of N due to N immobilization and mineralization. When the difference in soil total N between the final and initial level is positive, the change represents N sink in the soil due to addition of inorganic N fertilizers, organic manure, and amendments and conversion of inorganic N to organic form as a result of increased N immobilization. The reverse is true for N source when the value is negative, a result of increased mineralization of crop residue and soil organic N to inorganic N forms which are either taken up by the crop or lost to the environment. Some of these include residual soil N at crop planting and harvest and N mineralization potential of the soil during the growing season. To determine soil total N, soil samples are collected from various places within a treatment, composited, air-dried, and ground to 2 mm, from which a subsample is used for N analysis. If samples are collected at multiple depths, then samples from various places are composited by depth and prepared as above. The depth of the soil sample for N sequestration depends on treatments, management practices, soil and climatic conditions, and duration of the experiment. For example, in the no-till system, N sequestration may occur at the thin soil surface compared with the conventional till system where residue incorporation to a greater depth may increase N storage at the subsurface layer. For perennial cropping systems, N sequestration may occur further at greater depths due to increased root growth compared with annual cropping systems. Soil total N concentration can be determined either by using the Kjeldahl or Dumas method. The Dumas method, where soil sample is ignited at high temperature, provides better values of N concentration with <±5% uncertainty [3]. The Kjeldahl method is a wet oxidation method of total N analysis where soil sample is digested with H_2_SO_4_ and Na_2_SO_4_. The method has been used widely in the past, but disadvantages of the method are: (1) it takes long time to analyze soil samples, (2) it does not take account of NO_2_-N, NO_3_-N, and compounds containing N—N and N—O linkages for which pretreatment of the sample is required before N anallysis, and (3) it involves handling of hazardous chemicals [3]. The Dumas method is a dry oxidation method where the sample is ignited with CuO at high temperature in an induction furnace and released N_2_ is measured. The advantages of the Dumas method are: (1) determination of total N in samples is rapid, (2) it takes cares of all N fractions not accounted for by the Kjeldahl method, and (3) it consistently provides higher values of total N concentration compared with the Kjeldahl method [3]. The disadvantages of the Dumas method are: (1) the instrument can only analyze small amount of sample (50 mg of soil or 15 mg of plant sample) and (2) the sample needs to be finely ground (<250 μm) before analysis [3]. Because of the high spatial variability of soil total N, it is recommended that the experiment be conducted for at least five years to obtain significant changes in soil total N between initial and final levels [4]. The bulk density of the soil layer also needs to be determined for converting soil total N concentration (g N kg^−1^) to content (kg N ha^−1^). This is measured at the time of soil sample collection as the weight of oven-dried soil at 105 °C divided by the volume of the core. When soil samples are collected at various depth intervals, the equivalent soil mass method [5], which uses reference soil mass at the beginning of the experiment, is often used to calculate soil total N content at various depths at the end of the experiment.

#### Procedure

Determine soil total N concentration in samples using the procedure shown by Bremner [3]. Determine bulk density of the soil layer using the core method [6]. Calculate soil total N content using the following formula:

(5) STN = STN_c_ × BD × T × 10,000

Where STN = soil total N content (kg N ha^−1^), STN_c_ = soil total N concentration (g N kg^−1^), BD = bulk density (Mg m^−3^), T = thickness of the soil layer (m), and 10,000 = conversion factor. Calculate soil total N content for samples collected both at the beginning and end of the experiment. Use the equivalent soil mass method [5] to adjust the mass of soil during calculations of bulk density and soil total N content for each soil layer as a result of the effect of management practice and time. For the whole soil profile N content, add values of soil total N content from individual layers.

### Nitrogen inputs

#### Nitrogen fertilizer

Nitrogen fertilizer is the single most source of N applied in large amounts to enhance crop yield and quality. Nitrogen fertilization rates depend on crop species, crop N demand, and soil and climatic conditions among locations. Nitrogen fertilization rates to same crops also depend on crop varieties and differ from one location to other due to variations in residual soil N content to a depth of 60 cm at crop planting and N mineralization potential of the soil during the growing season. A depth of 60 cm is chosen to determine soil residual N content because it has been assumed that crop roots usually grow to that depth for N uptake [6], [7], [8]. This often results in analysis of soil samples for NO_3_-N content and N mineralization potential before crop planting. Both of these measurements are used to adjust N fertilization rates to crops. While soil NO_3_-N content can be directly measured by using chemical analysis, it is often difficult to measure N mineralization potential. One way is to estimate N mineralization potential by assuming that 1–2% of soil organic N is mineralized every year, which has uncertainty values of ±25 to 50% [7]. When soil tests are not done for NO_3_-N content, application of N fertilizers at recommended N rates exceeds crop N demand, resulting in increased accumulation of residual soil N and N losses through leaching, denitrification, and gas emissions.

##### Procedure

Determine N fertilization rates applied to crops by deducting soil NO_3_-N content and N mineralization potential to a depth of 60 cm before planting from desired N rates. For measuring soil NO_3_-N content, collect soil samples to a depth of 60 cm from several locations within a treatment in the field, composite, air dry, and ground to 2 mm. Determine NO_3_-N and NH_4_-N concentrations as suggested by Mulvaney [9] and total N concentration and bulk density as shown above. Calculate NO_3_-N, NH_4_-N, and total N contents by multiplying their concentrations by the bulk density and the thickness of the soil layer, similar to shown in Eq. (5). Calculate N fertilization rate to crops as:

(6) N_a_ = N_r_ − (NO_3_-N + 0.01 × [STN − NH_4_-N − NO_3_-N])

Where N_a_ = N fertilization rate (kg N ha^−1^), Nr = recommended or desired N fertilization rate to crops (kg N ha^−1^), and STN, NH_4_-N, and NO_3_-N = soil total N, NH_4_-N, and NO_3_-N contents (kg N ha^−1^) at 0–60 cm, respectively. Eq. (6) assumes that only 1% of soil organic N is mineralized during the growing season [7]. For soils containing high soil organic matter under irrigated cropping systems, it can be assumed that 2% of soil organic N will be mineralized. If the value of soil organic N is not available or it is not feasible to conduct soil analysis due to time constraint and NH_4_-N content is negligible, use only soil NO_3_-N content to calculate N fertilization rate. For N fertilization rates not adjusted to soil N contents, use recommended N rates applied to crops. The accuracy of N fertilization rate is ±5 to 10% of applied N. The uncertainty values can be reduced by intensive soil sampling from various locations within the plots, compositing, and testing samples for soil residual and mineralizable N at crop planting.

#### Manures and amendments

Manures and amendments containing N, such as animal manure, compost, crop residue, biochar, etc., with or without supplemental inorganic N fertilizer are sometime applied to enhance soil fertility and crop yield. In such cases, total N concentration in manures and amendments should be determined before applying them in the field. The rates of manures and amendments applied to crops are usually based on available N and P and crop N and P demand. For example, it has been determined that only 60–70% of manure N is available to plants during a growing season, while the rest is either converted into soil organic N or lost through volatilization, denitrification, leaching, surface runoff, and soil erosion [10]. The rates are also adjusted to soil residual NO_3_-N and N mineralization potential, similar to that observed for synthetic N fertilizers above. When manures are not analyzed for N concentration, the concentration can be estimated for various animal manures [11]. This, however, increases the uncertainty of manure N input, which ranges from ±30 to 50%. Another uncertainty arises from N loss due to NH_3_ volatilization. The loss ranges from 2 to 35% of applied amount, depending on manure and amendment type and application method [11]. The loss can be minimized by incorporating manures and amendments into the soil immediately after application, as surface application and retaining the manure in the soil for a longer period can increase the loss [12].

#### Biological nitrogen fixation

Nitrogen can be fixed symbiotically in legumes in association with Rhizobium bacteria. Because of the complex nature of legume-rhizobium symbiosis, relatively few information is available about the transfer of atmospheric N to plants through fixation. The amount of fixed N depends on legume species, available soil N content, crop management, soil water content, type of bacteria, and soil chemical properties [13]. As a result, the quantity of N fixation is usually estimated by legume N uptake which is determined by multiplying N concentration by total aboveground (stems, leaves, and grain) biomass yield. The accuracy of such value is ±10 to 20% of legume N. Because legumes can also take up N from the soil, N fixed in legumes is calculated by using a conversion factor which ranges from 65 to 75% of legume N uptake, depending on soil inorganic N content [1], [2]. It has been known that legumes fix maximum N when soil inorganic N is low [13]. Only those values of N fixation that are returned to the soil through crop residues and roots are considered for the calculation of N balance. Harvested grain and/or biomass N are excluded. As data on root N are rare, it is estimated that belowground biomass N constitutes about one-third of the total aboveground biomass (grains + stems + leaves) N [11]. Biomass N content is calculated by multiplying grain and/or biomass yield by N concentration in each component. Nitrogen fixation for legume in legume-nonlegume mixture is calculated by determining the percentage of legume in the mixture. The uncertainty in the estimation of legume N fixation is ±20 to 40% of total N fixed, depending on crop species and soil and climatic conditions. The uncertainty in N fixation is caused by differences in the ability of crop species or different cultivars of the same species to fix N as a result of varying association of root nodules with N-fixing bacteria, such as *Rhizobium* sp. Differences in N fixing ability of legumes also depend on seeds inoculated with or without *Rhizobium* sp. Furthermore, differences in the amount of crop biomass production due to variations in climatic conditions from year to year also affect the amount of N fixed in legumes.

Nitrogen can also be fixed nonsymbiotically by blue-green algae and free-living soil bacteria. The amount of N fixation depends on soil organic matter, water, and inorganic N contents, pH, and P and Mo levels [14]. In agroecosystems, nonsymbiotic N fixation is small and ranges from 3 to 7 kg N ha^−1^ yr^−1^ [2], [14].

##### Procedure

For symbiotic N fixation, determine grain and biomass (stems and leaves) yields of fresh legumes. Calculate dry matter yield on subsamples by drying them at 65 °C and determine yield on dry matter basis. Determine total N concentration (g N kg^−1^) in each plant component by using the method as shown by Bremner [3]. Find N content (kg N ha^−1^) by multiplying dry yield by N concentration. Assuming that 70% of legume N is biologically fixed [1], [2], [11], biological N fixation (kg N ha^−1^), when only grain N is removed from the soil, is calculated as:

(7) Biological N fixation = 0.7 × (Biomass N + 0.33 × total aboveground biomass N)

Where total aboveground biomass N refers to the sum of N contents in grain and biomass. The value 0.33 × total aboveground biomass N refers to belowground biomass N. When total aboveground biomass N is removed that also includes forage legume, biological N fixation is calculated as:

(8) Biological N fixation = 0.7 × 0.33 × total aboveground biomass N

For legume cover crops and green manure crops where total aboveground biomass N is returned to the soil, biological N fixation is calculated as:

(9) Biological N fixation = 0.7 × 1.33 × total aboveground biomass N

#### Irrigation water nitrogen

Nitrogen is also added from irrigation water applied to crops. Nitrogen concentration in water varies from one location or source to other and over time. It is determined by chemical analysis for total (inorganic + organic) N using Kjeldahl procedure [3]. Nitrogen input for irrigation water is calculated by multiplying total N concentration by the amount of water applied in the field. The accuracy of the measurement varies from ±5 to 15% of the total applied water N input. The uncertainty in the measurement is caused by spatial and temporal variations in N concentration of water flowing through streams and rivers from various sources. For dryland cropping systems, the value can be ignored.

#### Atmospheric nitrogen deposition

Nitrogen can also be added from precipitation (rain and snow). Nitrogen concentration in precipitation can be higher in regions with the local source of ammonia, such as cattle feedlot, barnyard, or poultry house. In USA, N input from precipitation usually ranges from 2 to 15 kg N ha^−1^ [11]. For accurate measurement, the value can be determined by multiplying average total N concentration in precipitation water by total annual precipitation for the area. For this, total N concentration in water collected during precipitation period is determined by using the Kjeldahl method, similar to the irrigation water above. Another atmospheric deposition is dry deposition which results from absorption of ammonia and other compounds from the atmosphere to the field. The value also varies from one location to the other, with higher values near the local ammonia source. The estimated N input for dry deposition is similar to that for precipitation N [10]. The uncertainty of both wet and dry deposition of atmospheric N varies from ±5 to 15% of the total N deposition. The uncertainty primarily results from the distance of the site from ammonia source, such as livestock barnyards and farms where high rates of ammonia-based N fertilizers are applied to crops.

#### Seed nitrogen

Seed used for planting crops can add N to the soil [1], [2]. The value for seed N can be determined by multiplying N concentration in the seed by seeding rate. The uncertainty of such value is ±10 to 20% of the applied seed N. The uncertainty is due to variations in N concentration of seeds of various crop species and various cultivars of same species.

### Nitrogen outputs

#### Crop nitrogen removal

Crop N removal includes N removed in harvested aboveground plant components, such as grain and/or biomass, from a known area in the field. For cereal crops, only grain is harvested. If both grain and straw are harvested, then crop N removal includes both grain and straw N. For forage crops, crop N removal includes N in harvested aboveground biomass. For a multi-year experiment, N removal is determined in every year and total N removal is determined by adding N removal in each year.

##### Procedure

Determine crop grain and/or biomass yield at harvest from a known area in the field by weighing. Crop is harvested either by hand or using a machine, such as a plot combine. The harvestable area depends on the size of the plot. Determine dry matter yield by drying a sample at 65 °C for 3–7 d and convert yield to dry matter basis. Grind a subsample of grain and/or biomass to <1 mm and determine N concentration by using methods similar to soil samples as described by Bremner [3]. Determine crop N removal (kg N ha^−1^) by multiplying crop yield by N concentration. The uncertainty in crop N removal ranges from ±15 to 25% of total N uptake, depending on crop yield. The uncertainty is caused by variations in N concentration and crop yield from year to year due to differences in climatic conditions, such as air temperature and precipitation, and soils that have variable N concentrations. For example, crops grown in soils with higher N concentration will have more N removal due to greater yield than soils with lower concentration. The uncertainty can be reduced by increasing the size of the harvestable area and using the proper method of analyzing N concentration in the plant sample [3], [11].

#### Ammonia volatilization

Nitrogen from fertilizers and manures can be lost to the atmosphere through ammonia volatilization. The loss depends on the type of fertilizer and manure, method of application, N rate, soil pH, texture, and cation exchange capacity, and climatic condition. For manures, N loss also depends on N concentration of manure and duration of application. Nitrogen loss ranges from 10 to 20% of applied N for inorganic N fertilizers and 15 to 30% for manures [1], [12]. Nitrogen loss through ammonia volatilization can be measured in the field using complex experiments [15]. Because of the complexity and difficulty in measuring ammonia volatilization, the values can also be estimated for various N sources, soil conditions, and management practices [16]. The accuracy of such measurements and estimates ranges from 20 to 50% of applied N [16]. The uncertainty in the values mainly results from variations in N sources and soil and climatic conditions as stated above. Ammonia volatilization from biologically fixed N is considered negligible.

#### Denitrification

Denitrification occurs in anaerobic condition when NO_3_-N is converted into N_2_ gas by denitrifying bacteria. During low oxygen level in the soil, denitrifiers use NO_3_-N rather than O_2_ for microbial respiration. The process usually occurs when soil water content increases during irrigation and/or precipitation. Denitrification loss of N depends on soil temperature, water content, organic matter, inorganic N level, and pH. Denitrification loss can be measured in the field using several methods [15], [17]. Such methods are often complex and time consuming. In such cases, denitrification loss can be estimated for soils with various organic matter contents and drainage conditions, and the value is adjusted for applications of tillage, manure, and irrigation, cropping system, soil bulk density, climatic condition, and ammonia loss through volatilization [11].

##### Procedure

Determine N loss through denitrification by using procedures reported by several researchers [15], [19]. When denitrification loss cannot be measured due to complexity and physical constraints, the loss can be estimated for soil with different organic matter contents and drainage conditions [11]. For determining N loss from inorganic N fertilizer and manure, deduct N loss through ammonia volatilization from N fertilization rate and find total N input by adding inputs from N fertilizer, wet and dry depositions, and irrigation. Note that N losses through ammonia volatilization from inorganic N fertilizer and manure are different and denitrification loss of N from manure is twice as that from N fertilizer. If both inorganic N fertilizer and manure are applied together, then calculate denitrification N loss separately for each component and find the total loss by adding them. The uncertainty of denitrification N loss ranges from ±20 to 50% of the applied N. The uncertainty in the measurement results from variations in soil and climatic conditions, drainage conditions, and management practices. The uncertainty can be reduced by using accurate methods of measuring denitrification, such as the ^15^N isotope method [17].

#### Nitrogen losses through soil erosion, surface runoff, gas emissions, and plant senescence

Nitrogen losses through soil erosion, surface runoff, gas emissions, and plant senescence are small and can often be ignored. Nitrogen loss through soil erosion by water primarily occurs in sloping land and depends on soil texture, length and steepness of slope, conservation practices used, amount of soil lost, N content of the soil, and climatic condition [1], [2]. Soil erosion by wind can also result in N loss, primarily in arid and semiarid regions. Nitrogen loss through soil erosion is estimated by multiplying the amount of annual soil loss by soil organic matter content and 2 (conversion factor) [18].

Nitrogen loss through surface runoff depends on the extent of soil cover by plants, source of N applied, precipitation intensity, and soil crusting. Such loss usually amounts to <3 kg N ha^−1^ yr^−1^ [18]. Gaseous loss of N (other than NH_3_ volatilization which is explained above) occurs as NO_x_ emissions, out of which N_2_O is a potent greenhouse gas that contributes to global warming. Emissions of NO_x_ can be measured by using various techniques, such as static and dynamic chambers and micrometerological methods [15], [19]. If measurements cannot made, NO_x_ emissions can be estimated as 1–2% of applied N input [20]. Using such estimated values to calculate the N balance, however, adds uncertainty. Improved management practices, such as reducing N fertilization rates by including legumes in the crop rotation, applying polymer-coated N fertilizers, and incorporating fertilizers and manures into the soil immediately after application, help to reduce gaseous N losses [19]. Nitrogen loss through plant senescence occurs as ammonia and volatile amines emissions. Such loss ranges from 2 to 8% of aboveground plant N (or about 4 to 18 kg N ha^−1^ yr^−1^) [11].

#### Nitrogen leaching

Nitrogen leaching occurs when N input exceed crop N demand, when N fertilizer is applied during low crop demand, and when residual soil N content is high. Leaching occurs especially in irrigated cropping systems and in regions with humid climate and coarse-textured soils. Nitrogen loss through leaching can range from 5 to 50% of applied N input [21]. Some researchers [2], [11] have found that N leaching loss can range from 12 to 75 kg N ha^−1^, depending on crop types, cropping system (irrigated or dryland), soil texture, N fertilization rate, and climatic condition. Nitrogen leaching can be measured by using lysimeters, deep soil sampling, and soil solution sampling with porous cups, but such measurements require detailed study over a number of years [21]. One of the ways to estimate N leaching is to observe for negative N balance using Eq. (1) when other parameters are known [1], [2]. Such estimated values, however, have large uncertainty, because N balance is calculated from the differences between N inputs and outputs where some parameters are also estimated, as explained above. Computer programs are now available to precisely estimate N leaching for various N rates and sources, crop types, cropping systems, management practices, and soil and climatic conditions [16]. Cover cropping, planting deep-rooted crops, such as perennial grasses, and synchronizing N application with crop N demand to increase N-use efficiency help to reduce soil residual N content and therefore the potential for N leaching.

## Additional information

A major nutrient applied in large amounts to sustain agricultural productivity and crop quality is N. Although N fertilization can increase crop yields, excessive application of N fertilizers can have detrimental effects on soil and environmental quality, such as increased soil acidification, N leaching, and emissions of NH_3_ and NO_x_ gases, out of which N_2_O is a highly potent greenhouse gas that contributes to global warming [1], [2]. Crops usually remove 50–60% of applied N, leaving the unused portion as residual soil N (NO_3_-N + NH_4_-N) after crop harvest [22]. Such residual N can be lost to the environment through leaching, denitrification, surface runoff, soil erosion, and N_2_O emissions [2], [22]. Nitrogen loss to the environment can be minimized by increasing soil N storage using improved compared with traditional management practices [1], [2], [22].

Nitrogen cycling in agroecosystems is a complicated process [2], [11]. Although synthetic N fertilizers and manures constitute major portions of applied N in crop production systems, N is also added through dry and wet (rain and snow) depositions from the atmosphere, biological N fixation, and irrigation water. While N in crop grains and biomass during harvest represent major portions of N removal from agroecosystems, N is also lost to the environment through various processes. Unharvested N in crop residue (stems and leaves) and roots is recycled back to the soil, which forms the core of soil organic N. Accounting of all N inputs and outputs and retention in the soil provide an estimate of N balance that helps to optimize N availability for crop growth, increase N-use efficiency, and reduce N fertilization rate and the potential for N losses [1], [2], [23]. Nitrogen balance is a sensitive indicator of agroecosystem performance and environmental quality, especially for long-term experiments [2], [12].

Crop N requirements are usually determined by yields that are economically profitable, although yields may not be at their maximum levels [8]. Crop responses to N fertilization vary with soil NO_3_-N content and N mineralized from crop residue and soil organic N during the growing season. Maximum attainable yield for a crop vary with soil and climatic conditions, nutrient supply, and competitions with weeds and pests [8]. Therefore, it is necessary that soils be tested for residual N content and potential N mineralization (if possible) and adjusted for N rates before N fertilizers are applied so that N requirements for crops can be optimized and potential for N losses minimized.

Nitrogen balance in agroecosystems varies with soil and climatic conditions, crop species, and management practices [1], [2], [11]. This is due to differences in N fertilization rates, crop N removal, N mineralization and immobilization, atmospheric N depositions, N concentration in irrigation water, biological N fixation, and N losses through various processes among years and locations. Fine-textured soils retain more N and reduce N losses compared with coarse-textured soils, which can reduce N fertilization rates, although predominant N losses are gaseous losses and N leaching in fine-and coarse-textured soils, respectively [8], [14]. Soil and crop management practices, such as no-till and crop rotation, may result in different N fertilization rates to same or different crops, soil N retention, and N losses compared with conventional till and monocropping [23].

Nitrogen balance studies have been reported in several long-term studies [1], [2], [24]. Because of the complexity of measurement as well as time, labor, and cost constraints, some parameters have to be estimated which add uncertainty to calculated values. Some of the parameters used for calculating N inputs and outputs are either difficult to measure or need thorough and consistent measurements over time and space due to variations in soil and climatic conditions. Parameters, such as biological N fixation, atmospheric N depositions, and N losses through volatilization, denitrification, leaching, surface runoff, soil erosion, and gaseous emissions, need to be estimated due to various physical constraints for measurements, which create uncertainty in the calculation of N balance [1], [12]. Other uncertainties result from the measurements of parameters due to high spatial and temporal variability. Slow changes in soil total N content in short-term experiments can also add uncertainly in the calculation of N balance. Therefore, N balance should be calculated in long-term experiments (>5 yr).
